# “Phoenix Rising”: A scoping review on the impacts of dragon boating exercise on well-being among breast cancer survivors and factors affecting their participation

**DOI:** 10.1016/j.jesf.2026.200453

**Published:** 2026-01-23

**Authors:** Nelson Chun Yiu Yeung, Victor Chi Wing Tam, Stephanie Tsz Yung Lau, Lihua Pan, Sze Nok Ng, Deng Yau Shy, Raymond Kim Wai Sum

**Affiliations:** a The Jockey Club School of Public Health and Primary Care, Faculty of Medicine, The Chinese University of Hong Kong, Hong Kong, China; b Department of Physical Education, National Taiwan Normal University, Taiwan; c Department of Sports Science and Physical Education, Faculty of Education, The Chinese University of Hong Kong, Hong Kong, China

**Keywords:** Dragon boating, Breast cancer survivors, Well-being, Facilitators, Barriers, Physical activity

## Abstract

**Objective:**

Breast cancer survivors (BCS) often experience declines in physical and psychosocial well-being post-treatment, making supportive interventions essential. Dragon boating(team-based paddling in a long boat) has emerged as a promising, popular exercise for BCS. However, no comprehensive reviews exist on its impacts or participation factors. This scoping review addressed these gaps by examining two research questions (RQ): RQ1) the impacts of dragon boating on BCS’ well-being, and RQ2) facilitators and barriers influencing their participation.

**Methods:**

Seven databases (PubMed, Embase, CINAHL, Web of Science, PsycInfo, Scopus, CNKI) were used to search for eligible studies from January 1996 to November 2025 involving BCS engaging in dragon boating. Screening from 245 records, 33 articles (18 quantitative, 14 qualitative, 1 mixed-methods) were identified.

**Results:**

Among studies addressing RQ1 (n = 27), 24 studies reported at least one benefit on well-being. Dragon boating improved BCS’ physical functioning (e.g., upper limb strength, range of motion), psychosocial well-being(e.g.,mental health, posttraumatic growth), and health behaviors(healthier lifestyle), without increasing lymphedema risk. Benefits on biochemical indicators (e.g., anti-inflammatory markers/antioxidant capacity) were less conclusive. For RQ2 (n = 10), common facilitators for joining dragon boating were social support, focus away from cancer, information sharing, and being physically active/competitive; common barriers included feeling not fit enough to participate, reminders of death/cancer recurrence, concerns about cancer identity disclosure, time commitment, and location constraints.

**Conclusions:**

Most studies demonstrated the benefits of dragon boating for BCS' physical and psychosocial well-being, but evidence did not support its superiority to other activities. To maximize impacts, addressing facilitators/barriers of participation will be important when designing and implementing dragon boating programs for BCS.

## 1 Introduction

Breast cancer is the most commonly-diagnosed cancer among women worldwide, contributing to a significant burden of morbidity and mortality. With the advancements in diagnosis and treatment, breast cancer survivors (BCS) are having improved survival rates. However, research has shown that BCS are subject to decreased physical and psychosocial well-being,^1^ plus risks of developing lymphedema (i.e., a defect in the lymphatic system that causes limb swelling) due to removal of lymph nodes removal in breast cancer surgery or adjuvant therapies. At present, there is no cure for lymphedema but exercise may lower the risk of secondary lymphedema.^2^ These findings suggest that maintenance of physical and psychosocial functioning after breast cancer surgery is therefore important for better cancer survivorship.

Traditionally, BCS were suggested to avoid vigorous, repetitive upper body exercise, and excessive weight bearing on the side of their surgery.^3^ However, recent evidence has indicated that exercise interventions could have beneficial effects on the reduction of treatment-induced side-effects and improvements in overall well-being among BCS. A review of 29 studies has found that exercise interventions bring positive changes in multiple health outcomes (e.g., physical fitness, muscle strength, quality of life (QoL), fatigue, depression, anxiety, self-esteem) among postoperative BCS (even during adjuvant therapy).^4^ Another review of 26 randomized controlled trials (RCTs) showed that exercise interventions substantially improved QoL, physical functioning, and social functioning among BCS.^5^ In the same study, the meta-regression analysis results also indicated that exercise sessions with longer durations (60–90 min) improved cancer survivors' QoL to a greater extent than shorter exercise sessions.^5^ A recent intervention study in Ukraine found that BCS in group-based water-resistance exercise group reported significantly higher emotional well-being and fewer negative symptoms, compared to those participated in yoga and Pilates interventions.^6^ Such findings indicate the potential benefits of water resistance exercise interventions on BCS’ well-being.

Dragon boating is emerging as a potentially effective intervention suitable for BCS. Dragon boating is a water sport originated from China that involves a team of paddlers propelling a long, narrow boat with dragon-shaped heads and tails through the water. The physical demands of dragon boating require whole-body coordination, strength, and endurance, making it an ideal exercise for BCS’ rehabilitation after treatments. In the 1990s, the first BCS dragon boat team was established in Canada by McKenzie and found that weekly dragon boat training improved physical well-being among 24 BCS (including improved upper body strength, range of motions, and no lymphedema cases observed).^7^ Since then, more qualitative and quantitative studies emerged to demonstrate the health benefits of dragon boating exercise for BCS in other countries. Previously, Harris and colleagues^8^ published a narrative review on the potential benefits of dragon boating exercise for BCS’ health outcomes, but it did not systematically describe the methods and define the scope of the review (as studies on other types of physical activities were also reviewed). More recently, a systematic review of 10 quantitative studies by Herrero-Zapirain and colleagues^9^ also indicated that dragon boating interventions generally improved BCS’ quality of life and reduced disease-/treatment-related symptomatology. However, that review did not comprehensively review qualitative studies, summarize the impacts of dragon boating on non-self-reported health outcomes(e.g., biological indicators), and more importantly the unique facilitators and barriers associated with their participation in such exercise programs.

It has been suggested that healthcare professionals should also pay attention to motivators and barriers affecting BCS’ participation in physical activities.^10^ For example, a qualitative meta-synthesis of 13 studies reviewed the important barriers and facilitators associated with physical activity engagement among BCS.^11^ They identified the expected benefits on physical and psychosocial well-being, feeling of empowerment, having knowledgeable instructors, provision of tailored information, and supportive environment as the major facilitators, whereas physical discomfort (pain/fatigue) and other life responsibilities (work/family) as the major barriers. Given that dragon boating is a group-based paddling watersport with unique environmental factors and psychosocial/interpersonal dynamics, specific facilitators and barriers might contribute to BCS’ participation. Therefore, a comprehensive review will provide important information for healthcare providers to better design dragon boating exercise programs that benefit BCS’ multiple domains of well-being with the consideration of their facilitators and barriers for participation.

### 1.1 Purpose of the review

Given the growing interests in dragon boating exercise as a potential intervention for BCS, this scoping review aimed to fill the knowledge gaps by comprehensively reviewing existing research on the potential impacts of dragon boating on different domains of BCS’ health outcomes(including non-self-reported indicators (e.g., biological/physiological indicators), plus exploring the unique facilitators and barriers associated with BCS’ participation in dragon boating exercises.

## 2 Method

The review was informed by Arksey and O'Malley's^12^ scoping review framework, inclusive of the enhancements proposed by Levac et al.^12^ and Peters et al.^13^ scoping review process were adhered to, including (1)identifying the research questions, (2)identifying relevant studies, (3)study selection, (4)charting the data, and (5)collating, summarizing, and reporting the results.

### 2.1 Stage 1: Identifying the research questions

This scoping review aimed to answer the following research questions (RQ):

1. What has been found in the current literature regarding the impacts of dragon boating exercise on different dimensions of breast cancer survivors' well-being?
2. What are the facilitators and barriers associated with breast cancer survivors' participation in dragon boating?

A “wide” approach was employed such that a variety of dragon boating exercise programs for BCS were included in the review.

### 2.2 Stage 2: Identifying relevant studies

Next, articles were compiled through searching databases, hand-searching key journals, and relevant organizations, and checking reference lists. Consultation with the research team's institutional academic librarian was also conducted to develop, execute, and refine the search strategy. In accordance with Levac et al.^12^ and Peters et al.^13^ recommendations, the search strategy was piloted and continuously refined. We also included hand searching key journals and the reference lists of the selected articles.

The research team developed search terms aimed at examining the health impacts of dragon boating exercise programs among breast cancer survivors (RQ1) and the factors associated with participation in dragon boating exercise program among those survivors (RQ2). First, a preliminary search was conducted. The team then added search terms to the list, including new search terms found in the search results and adjustments to accommodate each database.

Seven databases (PubMed, Embase, CINAHL, Web of Science, PsycInfo, Scopus, CNKI) were used to search for eligible studies from January1996 (since the development of the first BCS dragon boat team in Canada) to November 2025. The search used the following search teams: (‘Breast cancer’ OR ‘breast carcinoma’ OR ‘mammary cancer’ OR ‘cancer of the breast’ OR ‘breast neoplasms’) AND (‘dragon boat∗’ OR “dragonboat∗”). The search was supplemented by reviewing the reference lists of the included articles.

### 2.3 Stage 3: Study selection

A study was included if it (a)involved BCS who have experience in dragon boating exercises; b)was a primary research report available in English or Chinese. A study was excluded if it (a)involved survivors of other cancer types in dragon boating exercises, (b)was not an empirical research study (e.g., a review/commentary/news article), (c)was a study protocol of a future study, (d)did not examine health impacts of dragon boating exercise on BCS or factors associated with participation with dragon boating.

Following Levac's recommendation,^12^ two reviewers (VT, SL) met to discuss inclusion criteria before independently reviewing abstracts and subsequent full texts. Any discrepancies during the review process were resolved with the third reviewer(i.e., the principal investigator).

Among the 245 articles that were identified and gathered from the databases, 165 of them were eliminated as duplicates. Among the remaining 80 articles, 30 were excluded during the first round of title and abstract screening. The reasons for exclusion were inappropriate publication type such as reviews, conference abstracts, magazines (n = 14), study design not relevant to dragon boating (e.g., rowing) (n = 8), target populations not specific to breast cancer (i.e., not targeted to breast cancer populations) (n = 6), foreign language publication(n = 2). We sought retrieval of full text of the remaining 50 articles, 6 were excluded due to inaccessible full-texts.

**Fig. 1:**
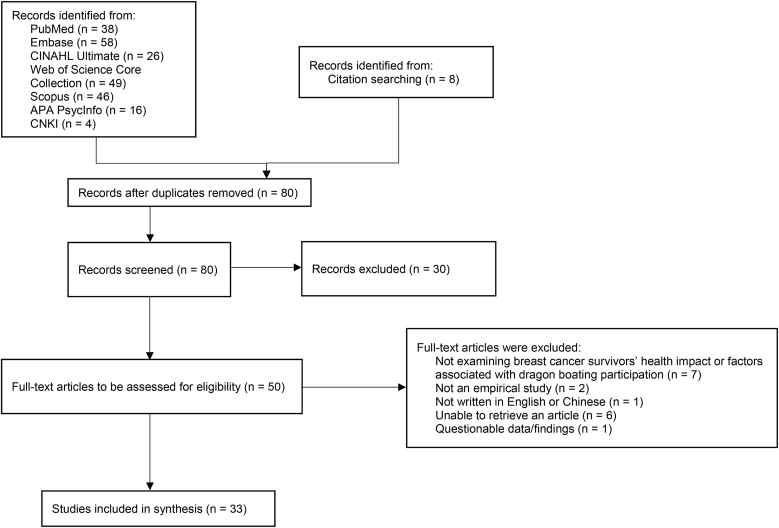
PRISMA flow diagram for scoping review.

Among the remaining 44 articles assessed for eligibility, 11 were excluded due to inappropriate publication type such as systematic reviews (n = 2), not examining BCS’ health impact or factors associated with dragon boating participation (n = 7), foreign language publication (n = 1), and questionable data/findings (n = 1). In addition, 8 articles were identified from citation searching. Therefore, 33 articles (27 for RQ1 and 10 for RQ2) were included in this review (Fig. 1). Data screening and management were supported by Rayyan.^14^ This review also evaluated the quality of the articles, even though Arksey and O'Malley^15^ regarded quality appraisal is optional in a scoping review.

### 2.4 Stage 4: Charting the data

An iterative approach to data charting was employed as recommendations from established guidelines.^12^^,^^13^ The reviewers independently extracted data for the first five included studies and refined the extracted conceptual headings to better align with the objective prior to completing extraction. Those data included the first authors, year of publication, information about the intervention programs/participants' dragon boating experience, and the major findings about the impacts of dragon boating experience on well-being/facilitators and barriers of dragon boating participation. Any disputes were dealt with through discussion until an agreement was reached. For RQ1, we considered different domains of well-being potentially impacted by dragon boating (e.g., physical functioning, biochemical indicators, psychosocial well-being, behavioral health). For RQ2, factors associated with BCS’ participation in dragon boating (including facilitators and barriers) were reviewed.

### 2.5 Stage 5: Collating, summarizing, and reporting results

Articles were analyzed using a combination of basic descriptive summary and deductive thematic analysis guided by predefined conceptual categories. An a-priori template, structured around the two research questions (RQ1 and RQ2), was developed to organize and guide the analysis process. Similar analytic strategies were also applied in previous scoping reviews in the context of cancer care.^16^ The scoping review was conducted in accordance with the recommendations of Preferred Reporting Items for Systematic reviews and Meta-Analyses extension for Scoping Reviews (PRISMA-ScR).^17^ The PRISMA-ScR checklist was also provided as a supplementary material (Supplementary Table 1).

To help the readers better interpret the findings, we also evaluated the selected studies’ quality guided by the Mixed Methods Appraisal Tool (MMAT).^18^ The MMAT was designed to critically appraise quantitative, qualitative, and mixed-methods studies included in reviews having multiple types of selected studies. The studies were independently evaluated by VT and SL; discrepancies were also resolved by the first author (NY). The MMAT allows the assessment of the appropriateness of the aim of the study, methodology, study design, participant recruitment, data collection, data analysis, and the findings presented. With five assessment items, the quality of studies was graded with a score ranging from ≤50 % as low quality, 51–75 % considered as an average quality, to 76–100 % considered as high quality. This appraisal tool has also been applied in other scoping reviews.^19^^,^^20^

## 3 Results

### 3.1 Study characteristics

**Fig. 2:**
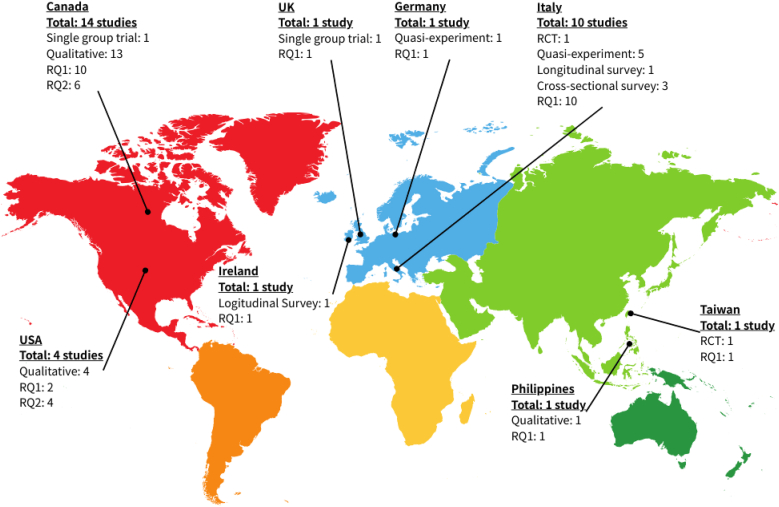
Map showing the geographic distribution of studies conducted to address the two research questions (N = 33) RQ1: What has been found in the current literature regarding the impacts of dragon boating exercise on different dimensions of breast cancer survivors' well-being? RQ2: What are the facilitators and barriers associated with breast cancer survivors' participation in dragon boating.?.

Among all the 33 articles identified, most of the studies were conducted in Canada (n = 14), Italy (n = 10), and the United States (n = 4). Others were conducted in Germany (n = 1), Ireland (n = 1), Philippines (n = 1), Taiwan (n = 1), and United Kingdom (n = 1) (Table 1). The geographic distribution of studies addressing RQ1 and RQ2 was also illustrated in a map (Fig. 2). All studies reported participants’ mean age as around 50–55 years old. The dragon boating exercise programs reported in the literature varied a lot. Among all studies, 27.3 % of dragon boating exercise programs lasted for 3 months of less; others lasted for 4–6 months (21.2 %) and more than 6 months (18.2 %). The number of sessions per week across the studies ranged from 1 to 4 sessions; 9.1 % of programs had one dragon boating exercise session per week; others had two and three dragon boating exercise sessions per week (18.2 % and 15.2 % respectively). The duration per session ranged from 60 to 180 min across the studies; 12.3 % of programs lasted for less than 2 h per dragon boating exercise session; others lasted for 2 h and 3 h per dragon boating exercise session (9.1 % and 3 % respectively) (Table 1).

**Table 1:** Characteristics of the selected studies (N = 33).

Study characteristics	Frequency/(%)
*Study design*	
Experimental (e.g., RCTs, quasi-experimental, single-group)	10 (30.3 %)
Observational (e.g., cross-sectional surveys, qualitative interviews)	23 (69.7 %)

*Type of data analyzed*	
Quantitative	18 (54.5 %)
Qualitative	14 (42.4 %)
Mixed-methods	1 (3.0 %)

*Research scope*§	
Impacts on well-being (RQ1)§	27 (81.8 %)
Physical functioning	17 (51.5 %)
Biochemical and biophysical indicators	6 (18.2 %)
Psychosocial well-being	18 (54.5 %)
Health behaviors	3 (9.1 %)
Facilitators and barriers of participation (RQ2)§	10 (30.3 %)

*Origin of study*	
Canada	14 (42.4 %)
Italy	10 (30.3 %)
USA	4 (12.1 %)
UK	1 (3.0 %)
Germany	1 (3.0 %)
Ireland	1 (3.0 %)
Philippines	1 (3.0 %)
Taiwan	1 (3.0 %)

*Participants having dragon boating experiences before the study*	
Yes	24 (72.7 %)
No	9 (27.3 %)

*Duration of dragon boating exercise program: duration*	
3 months or less	9 (27.3 %)
4–6 months	7 (21.2 %)
More than 6 months	6 (18.2 %)
Not explicitly stated	11 (33.3 %)

*Number of sessions per week in the dragon boating exercise program*	
Once	3 (9.1 %)
Twice	6 (18.2 %)
Three times	5 (15.2 %)
Not explicitly stated	19 (57.6 %)

*Number of hours per session in the dragon boating exercise program*	
1	2 (6.1 %)
1.5	2 (6.1 %)
2	3 (9.1 %)
3	1 (3.0 %)
Not explicitly stated	25 (75.8 %)

*Note*: §Several studies have included multiple outcomes or addressed both research questions; therefore, the number did not add up to 33.

For RQ1, 27 articles (10 experimental studies and 17 observational studies) explored the potential impacts/benefits of dragon boating on one domain in breast cancer survivors’ well-being, including physical functioning (17 studies), biochemical/biophysical indicators (6 studies), psychosocial well-being (19 studies), health behaviors (3 studies). Among the experimental studies (n = 10), different designs were used to evaluate the impacts of dragon boating exercise on well-being (2 RCTs, 6 quasi-experimental studies, 2 single-group trials). The duration of exercise programs ranged between 7 weeks and 4 years. The sample sizes for those experimental studies ranged between 16 and 105 (Table 1). Another 17 observational studies (5 quantitative studies, 11 qualitative studies, 1 mixed-methods study) explored the health outcomes among those who have already engaged in dragon boating exercises. For those quantitative studies, the duration of engaging in dragon boating exercise ranged from 6 months to 3 years. The sample sizes for those experimental studies ranged between 38 and 131. For qualitative/mixed-methods studies, most of them (n = 7) involved BCS participating in dragon boat programs in their first/second season, with sample sizes ranging between 3 and 100 (Table 2).

**Table 2:**
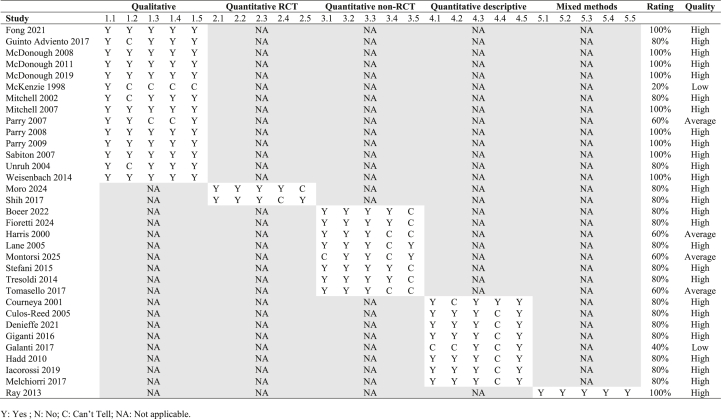
Quality assessment of included studies using the Mixed Methods Appraisal Tool (MMAT) (N = 33).

For RQ2, 10 studies (3 quantitative, 7 qualitative) explored the factors associated with breast cancer survivors’ participation in dragon boat exercises and shared stories about successful implementation of dragon boat exercise programs. All studies involved BCS who were also members of dragon boating teams. The sample sizes ranged between 3 and 470 in quantitative studies and between 3 and 20 in qualitative studies (Table 3).

**Table 3:** Characteristics of interventional studies exploring breast cancer survivors participating in dragon boat training programs (n = 10 studies).

	First author, year (region)	Design	Intervention	Major outcomes (Measurements)	
1	Moro, 2024 (Italy)	RCT (N = 31)	Intervention group (18 BCS): Dragon boat paddling exercise (1 h/session; 3 sessions/week for 12 weeks) Control group (13 BCS): 10 home-based 60-s exercises; repeated according to participants' feelings	Statistically significant	**Compare intervention and control:** Physical functioning: Improved lower limb strength (30CST); improved cardiorespiratory function (6MWT)

Not statistically significant	**Compare intervention and control:** Physical functioning: Shoulder mobility (ROM); upper limb strength (HDST) Biochemical and biophysical: Total body composition Psychosocial: QoL (SF-12); body image (BAS-2)
2	Shih, 2017 (Taiwan)	RCT (N = 60)	Intervention group (29 BCS): Dragon boat paddling exercise (3 h/session; 1 session/week for 8 weeks) + dragon boat race Control group (31 BCS): Not specified	Statistically significant	**Compare intervention and control:** Physical functioning: Improved upper limb strength (MMT)

Not statistically significant	**Compare intervention and control:** Physical functioning: Pain (VAS); shoulder mobility (ROM); lymphedema (ULC)
3	Boeer, 2023 (Germany)	Quasi-experiment (N = 98)	Intervention group (28 BCS): Dragon boat paddling exercise (1.5 h/session; 1 session/week for 8 weeks) Control group (70 BCS): Not specified	Statistically significant	**Pre-post training in the intervention group:** Physical functioning: Reduced lymphedema (ULC) Psychosocial: Improved QoL (EORTC QLQ-30)

Not statistically significant	**Pre-post training in the intervention group:** Psychosocial: QoL (SF-36)
4	Fioretti, 2024 (Italy)	Quasi-experiment (N = 29)	Intervention group (17 BCS): Dragon boat paddling exercise (1 h/session; 2 sessions/week for 6 months) Control group (12 BCS): Alternative physical activities (e.g., running, Pilates & Yoga) with personal preferences (>2 sessions/week)	Statistically significant	**Compare intervention and control:** Psychosocial: Decreased negative body image (BIS); reduced traumatic symptoms (IES-R)

Not statistically significant	/
5	Tresoldi, 2014 (Italy)	Quasi-experiment (N = 34)	Intervention group (20 BCS): 16-week exercise program: aerobic & resistance training (1 h/session; 2 sessions/week for 16 weeks) + dragon boat paddling exercise (1 h/session; 1 session/week for 8 weeks; start at week 8) Control group (14 BCS): sedentary patients	Statistically significant	**Pre-post training in the intervention group:** Biochemical and biophysical: Increased saliva anti-inflammatory markers, i.e., IL-6, IL-8, IL-15 (ELISA); improved biological antioxidant status (BAP test) **Compare intervention (pre-training) and control:** Biochemical and biophysical: Increased saliva anti-inflammatory markers, i.e., IL-8, IL-1ra (ELISA); improved oxidative status, i.e., serum hydroperoxides (photometry)

Not statistically significant	**Pre-post training in the intervention group:** Biochemical and biophysical: Saliva anti-inflammatory markers, i.e., IL-1ra, TNF-a (ELISA); oxidative status, i.e., serum hydroperoxides (photometry) **Compare intervention (pre-training) and control:** Biochemical and biophysical: Saliva anti-inflammatory markers, i.e., IL-6, IL-15, TNF-a (ELISA); biological antioxidant status (BAP test)
6	Tomasello, 2017 (Italy)	Quasi-experiment (N = 105)	Dragon boat group (25 BCS): Dragon boat paddling exercise (2 sessions/week for≥7 weeks) Walking group (25 BCS): Walking briskly outdoor (3–4 h/week) Breast cancer control group (25 BCS): No activities Healthy control group (30 health women): Not specified	Statistically significant	**Compare dragon boating and walking:** Biochemical and biophysical: Improved biological antioxidant activities (GP assay, SD assay) **Compare dragon boating and BCS control:** Biochemical and biophysical: Increased plasma ROS (colorimetric d-ROMs test); improved biological antioxidant status (BAP test); reduced plasma LPO (spectrophotometry); improved biological antioxidant activities (TPTG spectrophotometry, GP assay, SD assay)

Not statistically significant	**Compare dragon boating and walking:** Biochemical and biophysical: Plasma ROS (colorimetric d-ROMs test); biological antioxidant status (BAP test); plasma LPO (spectrophotometry); biological antioxidant activities (TPTG spectrophotometry); DNA damage (ANCA); DNA repair capability (NERCT) **Compare dragon boating and BCS control:** Biochemical and biophysical: DNA damage (ANCA); DNA repair capability (NERCT)
7	Montorsi, 2025 (Italy)	Quasi-experiment (N = 30)	Intervention group (15 BCS): Non-competitive dragon boat race (1 race; 32 km) Control group (15 health women): 5 health women underwent a dragon boat competition together with the intervention group + 10 health women only performed measurements at rest	Statistically significant	**Pre-post race in the intervention group:** Physical functioning: Altered general sensation, i.e., tired, cold, agitation, fatigue (VAS); reduced pain (VAS) Biochemical and biophysical: Increased saliva anti-inflammatory markers, e.g., IL-6, IL-10 & TNF-α (ELISA); increased saliva ROS (EPRS); increased oxidative damage, i.e., urine 8-iso-PGF2α level (ELISA); increased appetite hormones, e.g., saliva ghrelin (ELISA); increased urine creatinine (chromatography); decreased serum sodium, potassium, chlorine, magnesium level (ISE) **Compare intervention and control:** Biochemical and biophysical: Improved biological antioxidant capacity (TAC assay)

Not statistically significant	**Pre-post race in the intervention group:** Biochemical and biophysical: Biological antioxidant capacity (TAC assay); urine NO metabolites, e.g., nitrite & nitrate (Griess reaction assay); urine neopterin (chromatography); uric acid (ISE); serum phosphorus, calcium (ISE) **Compare intervention and control:** All other outcomes, except biological antioxidant capacity
8	Stefani, 2015 (Italy)	Quasi-experiment (N = 55)	Intervention group (55 BCS): Non-competitive dragon boat race (2 h/session; 2 sessions/week for 4 years) + involved in seasonal competitions Control group (36 health women): Alternative physical activities (e.g., running, cycling, tennis, swimming) (2 h/session; 3 sessions/week for 4 years)	Statistically significant	**Compare intervention and control:** Biochemical and biophysical: Improved diastolic function and myocardial performance (echocardiogram)

Not statistically significant	/
9	Harris, 2000 (UK)	Single-group trial with pre-post test (N = 24)	Intervention group (24 BCS): Dragon boat paddling exercise (2 months) + dragon boat racing (not specified)	Statistically significant	/

Not statistically significant	**Pre-post exercise program:** Physical functioning: Lymphedema (ULS)
10	Lane, 2005 (Canada)	Single-group trial with pre-post test (N = 16)	Intervention group (16 BCS): 20-week exercise program: aerobic & resistance training (around 1 h/session; 3 sessions/week for 20 weeks) + dragon boat paddling exercise (1.5 h/session; 2 session/week for 12 weeks; start at week 8)	Statistically significant	**Pre-post exercise program:** Physical functioning: Increased arm circumference (ULS); increased arm volume (water displacement); improved upper limb strength (SBPT)
				Not statistically significant	/

*Note*: 30CST, 30-s chair stand test; 6MWT, 6-min walk test, ergometry; ANCA, alkaline and neutral Comet assay; BAP, biological antioxidant potential test; BAS, Body Appreciation Scale; BIS, Body Image Scale; ELISA, enzyme-linked immunosorbent assay; EORTC QLQ, European Organization for the Research and Treatment of Cancer Quality of Life Questionnaire; EPRS, electron paramagnetic resonance spectrometer; GP, glutathione peroxidase; HDST, handheld dynamometer strength tests; HW, health women; IES-R, Impact of Event Scale-Revised; ISE, ion-selective electrode determinations; LPO, lipid hydroperoxide; MMT, manual muscle testing test; NERCT, Nucleotide Excision Repair Comet Test; NO, nitrogen oxide; QoL, quality of life; ROM, range of motion test; ROS, reactive oxygen species; SBPT, supine bench press test; SD, superoxide dismutase; SF, Short Form Health Survey; TAC, trolox-equivalent antioxidant capacity assay; TPTG, total plasmatic thiol group; ULC, Upper limb circumference; VAS, visual analogue scale.

Regarding quality of the studies, 27 (81.8 %), 4 (12.1 %), and 2 (6.1 %) studies were evaluated as having high, average, and low quality respectively (Table 2). Therefore, most of the studies included were in high quality.

### 3.2 Research question 1: Impacts on well-being

**Fig. 3:**
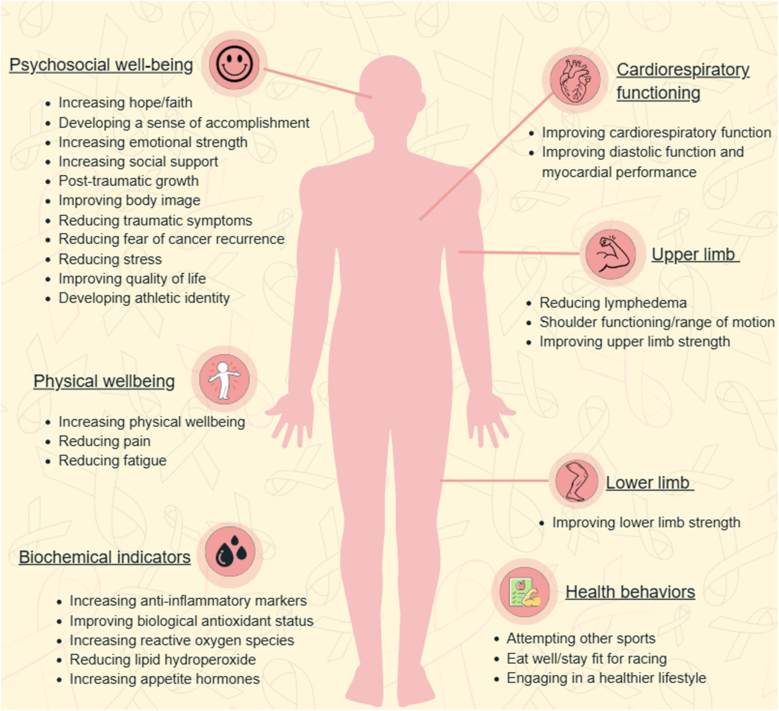
Summary of the benefits reported among breast cancer survivors after participating in dragon boating programs.

Overall speaking, among studies addressing RQ1 (n = 27), 24 studies reported at least one benefit on well-being. Dragon boating improved BCS’ physical functioning, biochemical/physiological indicators, psychosocial well-being, and health behaviors (Table 3, Table 4). There was no study reporting dragon boating increased BCS’ risk for lymphedema. Fig. 3 summarized the benefits reported among BCS after participating in dragon boating exercise program. Table 5 also integrated the findings from the studies addressing RQ1 regarding the impacts of dragon boating exercise program on different well-being indicators.

**Table 4:** Characteristics of observational studies (n = 17).

	First author, year (region)	Design	Dragon boating experience	Outcomes (Measurements)	
1	Denieffe, 2021 (Ireland)	Longitudinal survey (N = 38)	Dragon boat group (38 BCS): Participated in dragon boat racing in one season (March–October)	Statistically significant	**Pre-post dragon-boating season:** Physical functioning: Reduced fatigue (FACT-Fatigue); shoulder functioning (DASH)
				Not statistically significant	**Pre-post dragon-boating season:** Psychosocial: QoL (EORTC QLQ-30)
2	Iacorossi, 2019 (Italy)	Longitudinal survey (N = 100)	Dragon boat group (50 BCS): Participated in dragon boating at least 2 sessions/week for 6 months Other physical activities group (31 BCS): Participated in other physical activities at least 2 sessions/week for 6 months and/or underwent alternative treatment, e.g., compression therapy on lymphatic drainage	Statistically significant	**Pre-post 6-month dragon boating in the intervention group:** Physical functioning: Reduced physical symptoms, e.g., fatigue, pain, dyspnea, insomnia (EORTC QLQ-30); improved physical functioning (EORTC QLQ-30) Psychosocial: Improved overall QoL (EORTC QLQ-30); improved role, emotional and cognitive functioning (EORTC QLQ-30)
				Not statistically significant	**/**
3	Galanti, 2017 (Italy)	Cross-sectional study (N = 131)	Dragon boat group (55 BCS): Participated in dragon boating training (2 h/session; 3 sessions/week; at least 3 years) Prescribed exercise group (33 BCS): Participated in prescribed other exercise at least 3 sessions/week for at least 3 years Prescribed exercise group-healthy women (20 healthy women): Participated in prescribed exercise: fast walking (at least 30 min/session, 3 sessions/week) + strength training Athlete group-healthy women (23 healthy women) Participated in other physical activities, e.g., running, tennis, cycling, swimming (>2 h/session; 3 sessions/week)	Statistically significant	**/**
				Not statistically significant	**Compare among the 4 groups:** Physical functioning: Diastolic function and myocardial performance (2DSTE)
4	Giganti, 2016 (Italy)	Cross-sectional study (N = 66)	Dragon boat group (17 BCS): Participated in dragon boating training (2 h/session; 1 session/week) Sedentary group (15 BCS) Other physical activities group (14 BCS): Participated in other physical activities, e.g., treadmill, cycling, aerobic dance, + strength training (1 h/session; 3 sessions/week) Healthy other physical activities group (10 healthy women): Same exercise as above Healthy sedentary group (10 healthy women)	Statistically significant	**/**
				Not statistically significant	**Compare dragon boat group with the other 3 groups:** Biochemical and Biophysical: Serum level of MMP-2 & −9 (ELISA)
5	Melchiorri, 2017 (Italy)	Cross-sectional study (N = 64)	Dragon boat group (15 BCS): Other physical activities group (16 BCS): Sedentary group (16 BCS) Control group (17 healthy women)	Statistically significant	**Compare BCS and healthy women:** Psychosocial: QoL (SF-36)
				Not statistically significant	**Compare BCS and healthy women:** Physical functioning: Shoulder functioning (Constant score, Rowe score, DASH)
6	Mitchell, 2007 (Canada)	Qualitative Interviews (pre/post)	10 BCS: New members of a dragon boat team, participating in one season of dragon boating racing.		Physical functioning: Increased physical strength Psychosocial: Increased hope Developed an athlete identity Developed a sense of accomplishment Increased emotional strength Increased social support Post-traumatic growth: regain control of life, move forward to try new things, gain power to success Reduced fear of cancer recurrence
7	McDonough, 2008 (USA)	Qualitative Interviews (pre/post)	14 BCS: Participate in a dragon boat program in their first year		Physical functioning: Increased physical strength and energy Psychosocial: Developed an athlete identity Improved body image: physical appearance Increased social support Behavioral: Attempted other sports (transfer of skills from DB to other physical activities)
8	McDonough, 2011 (USA)	Qualitative Interview for 5 times	17 BCS: Attended at least one practice of a newly-formed dragon boating team and participated in first two seasons of dragon boating		Psychosocial: Increased social support Post-traumatic growth: appreciation of life, find new possibilities and opportunities, increase personal strengths, reinforced faith
9	McKenzie, 1998 (Canada)	Qualitative	24 BCS: Members of a newly formed dragon boat team Attending three training workouts per week and participating a dragon boat racing in the Vancouver festival		Physical functioning: None of the participants developed a new episode of lymphedema Improved range of motion (ROM) of shoulders Psychosocial: Developed a sense of accomplishment Increased social support
10	Parry, 2008 (Canada)	Qualitative Interview	11 BCS: Members of a dragon boat racing team (5 BCS were in their first or second season of dragon boating)		Physical functioning: Improved physical health Increased energy levels Psychosocial: Increased social support Enhanced stress coping skills Post-traumatic growth: foster appreciation
11	Parry, 2009 (Canada)	Qualitative Interview	11 BCS: Members of a dragon boat racing team (5 BCS were in their first or second season of dragon boating)		Psychosocial: Post-traumatic growth: find new purpose and meaning in life, foster empowerment, increase confidence to face life and death Increased social support
12	Sabiston, 2007 (Canada)	Qualitative Interview	20 BCS who had participated in dragon boating for 2–8 years		Physical functioning: Improved physical health Psychosocial: Developed an athlete identity Improved mental health Enhanced stress coping skills Increased social support Post-traumatic growth: appreciation for life, find new possibilities, increase psychological strengths
13	Unruh, 2004 (Canada)	Qualitative Interview twice	3 BCS: Involved in dragon boat racing within 6 months to 3 years following their cancer diagnosis Practice 3 times a week and attended various festivals during racing season, also with regular physical activity and monthly meetings		Physical functioning: Enhanced physical wellbeing Psychosocial: Developed individual and team pride Enhanced emotional wellbeing Increased social support Behavioral: Perceived a need to eat well and stay fit for racing
14	Mitchell, 2002 (Canada)	Qualitative Interview	6 BCS active members in dragon boat teams		Psychosocial: Increased hope Increased social support Post-traumatic growth: regain control of life, acceptance of breast cancer, able to move on, embracing life
15	Parry, 2007 (Canada)	Qualitative Interview	12 BCS (1 male; 11 female) involved in dragon boat racing team (5 BCS were in their first or second season of dragon boating)		Psychosocial: Developed an athlete identity Increased social support Post-traumatic growth: creating new life Relieved stress
16	Guinto-Adviento, 2017 (Philippines)	Qualitative Focus group discussion	3 BCS who participated in dragon boat paddling		Physical functioning: Increased physical strength Psychosocial: Increased family support Increased social support Increased emotional strength Post-traumatic growth: appreciation of life, acceptance of breast cancer, help dealing with cancer diagnosis and life, regain control of life and body
17	Ray, 2013 (Canada)	Mixed method Cross-section survey and interview	100 BCS: Active members for the dragon boat paddling session, n = 15 participated in the qualitative interview		**Quantitative findings:** Psychosocial: Developed faith (FACT-Spiritual) Improved QoL (FACT-General, FACT-Breast) **Qualitative findings:** Psychosocial: Increased hope Increased social support Post-traumatic growth: appreciation of life Behavioral: Engaged in healthier lifestyles

*Note*: 2DSTE, 2D speckle tracking echocardiogram; DASH, Disability of the Arm Shoulder and Hand; ELISA, enzyme-linked immunosorbent assay; EORTC QLQ, European Organization for the Research and Treatment of Cancer Quality of Life Questionnaire; FACT, Functional Assessment of Chronic Illness Therapy; MMP, matrix metalloproteinases; SF, Short Form Health Survey; ULC, Upper limb circumference.

**Table 5:** Summary of findings about the well-being outcomes studied (n = 27).

Indicators	Number of articles	Significant improvements [n(%)]	Non-significant changes [n(%)]
**Physical functioning**			
Improving lower limb strength	**1**	1 (100)	0
Improving cardiorespiratory function	**1**	1 (100)	0
Improving diastolic function and myocardial performance	**2**	1 (50)	1 (50)
Reducing lymphedema	**4**	1 (25)	3 (75)
Shoulder functioning/range of motion	**5**	2 (40)	3 (60)
Improving upper limb strength	**3**	2 (66.6)	1 (33.3)
Increasing physical wellbeing	**5**	5 (100)	0
Reducing pain	**3**	2 (66.6)	1 (33.3)
Reducing fatigue	**3**	3 (100)	0
**Biochemical**			
Increasing anti-inflammatory markers	**3**	2 (66.6)	1 (33.3)
Improving biological antioxidant status	**5**	3 (60)	2 (40)
Increasing reactive oxygen species	**3**	2 (66.6)	1 (33.3)
Reducing lipid hydroperoxide	**2**	1 (50)	1 (50)
Increasing appetite hormones	**1**	1 (100)	0
Total body composition	**1**	0	1 (100)
Oxidative status	**1**	0	1 (100)
DNA damage/repair capability	**1**	0	1 (100)
Biological antioxidant capacity	**1**	0	1 (100)
Urine nitrogen oxides metabolites/neopterin/uric acid	**1**	0	1 (100)
Serum phosphorus and calcium	**1**	0	1 (100)
Serum level of matrix metalloproteinases −2 & −9	**1**	0	1 (100)
**Psychosocial**			
Increasing hope/faith	**4**	4 (100)	0
Developing a sense of accomplishment	**2**	2 (100)	0
Increasing emotional strength	**3**	3 (100)	0
Increasing social support	**12**	12 (100)	0
Post-traumatic growth	**9**	9 (100)	0
Improving body image	**3**	2 (66.6)	1 (33.3)
Reducing traumatic symptoms	**1**	1 (100)	0
Reducing fear of cancer recurrence	**1**	1 (100)	0
Reducing stress	**3**	3 (100)	0
Developing athletic identity	**4**	4 (100)	0
Developing personal and team pride	**1**	1 (100)	0
Improving quality of life	**5**	4 (80)	1 (20)
**Health Behaviors**			
Attempting other sports	**1**	1 (100)	0
Eating well/staying fit for racing	**1**	1 (100)	0
Engaging in healthier lifestyle	**1**	1 (100)	0

### 3.3 Impacts of physical functioning

Among the 17 studies exploring impacts on physical functioning (10 quantitative and 7 qualitative studies), 15 of those reported at least one benefit of dragon boating in BCS’ physical functioning (including upper/lower limb strength, pain, fatigue, dyspnea, insomnia, shoulder functioning, cardiorespiratory function, and risks of lymphedema) (Table 3, Table 4).

Most studies demonstrated an increase in upper limb strength, which was assessed by manual muscle testing test^21^ and supine bench press test.^22^ However, Moro et al.^23^ revealed a non-significant improvement in upper limb strength (handheld dynamometer strength test), but significant lower limb strength (30-s chair stand test) in a randomized controlled trial (RCT) with 31 BCS.

Mixed results were found in pain reduction. One RCT^21^ did not find significant reductions in pain (measured by visual analogue scale; VAS) between the intervention and control groups. The impact on pain reduction emerged with a longer course of training duration (i.e., after a 6-month dragon boating), as revealed in a longitudinal observational study.^24^ Iacorossi et al.^24^ also reported improvements in BCS’ physical symptoms(e.g., fatigue, dyspnea, insomnia, and overall physical functioning).

Current evidence did not provide consistent support that dragon boating could improve shoulder mobility and cardiorespiratory/myocardial functions in BCS.^21^^,^^23^ Three observational studies (2 quantitative, 1 qualitative) reported inconclusive results on the effects of dragon boating in shoulder functioning. Denieffe et al.^25^ demonstrated improved DASH scores in 38 BCS participating in dragon boat racing for a season in Ireland; BCS in a qualitative study subjectively reported an improved range of motion of shoulders.^7^ However, Melchiorri et al.^26^ did not find significant differences in Constant score, Rowe score and DASH scores between the BCS dragon boating group and other BCS. On the other hand, a RCT with 31 BCS^23^ demonstrated a significant improvement in cardiorespiratory function from a 12-week dragon boat exercise program, but another quasi-experimental study in Italy^27^ did not find significant improvements in myocardial function using a 2D speckle tracking echocardiogram.

Regarding other indicators, there were 5 studies indicating that dragon boating did not increase the risk of lymphedema among BCS^7^^,^^21^^,^^28^^,^^29^ and 7 qualitative studies indicating that BCS perceived an increase in physical wellbeing^30^, ^31^, ^32^ and physical strength/energy.^3^^,^^33^^,^^34^

### 3.4 Impacts on biochemical and physiological indicators

Six quantitative studies (5 interventional studies, 1 observational) examined the changes in biochemical indicators after dragon boating exercises. All the five interventional studies compared between the intervention and control groups,^23^^,^^35^, ^36^, ^37^, ^38^ with two of those also compared the changes in biochemical indicators between pretest and posttest in the intervention group.^35^^,^^36^ The only observational study compared the levels of biochemical indicators across different groups of BCS and healthy women cross-sectionally.^39^ Among the 6 studies, 4 of them provided evidence suggesting the benefits of dragon boating in at least one biochemical and physiological indicator after participating in dragon boating (Table 3, Table 4).

Montorsi and colleagues^35^ reported a significant increase in anti-inflammatory markers (e.g., interleukin (IL)-6, IL-10, and TNF-α) but a non-significant decrease in biological antioxidant capacity among BCS in Italy after a non-competitive 32-km dragon boating race. However, those changes did not significantly favor the dragon boating group (compared to the control group). Similarly, Tresoldi et al.^36^ found that anti-inflammatory markers (e.g., interleukin (IL)-6, IL-8, and IL-15) and biological antioxidant capacity significantly improved among BCS after a 16-week dragon boat program. However, the change in oxidative damage was not significant. In a quasi-experimental study, Tomasella and colleagues^37^ found that the antioxidant capacity and oxidative stress (plasma reactive oxygen species (ROS) level) were significantly better among BCS participating in dragon boating (compared to BCS in the walking group and the no-activity group).

Other biochemical/physiological indicators were also examined in the reviewed studies (e.g., DNA damage and repair capacity,^37^ serum matrix metalloproteinases-2 (MMP-2),^39^ and serum matrix metalloproteinases-9 (MMP-9),^39^ and total body composition,^23^ but no significant improvements were apparent among BCS after dragon boating exercise.

### 3.5 Impacts on psychosocial well-being and health behaviors

Among 18 studies reporting findings on psychosocial impacts (6 quantitative, 11 qualitative, 1 mixed-methods), 17 studies supported at least one benefit of dragon boating in BCS’ psychosocial well-being. All qualitative and mixed-methods studies (n = 12) highlighted at least one psychosocial benefit, including improvements in mental health,^3^^,^^31^^,^^32^^,^^34^ stress coping skills,^31^^,^^40^^,^^41^ hope,^3^^,^^42^^,^^43^ fears of recurrence,^3^ body image,^33^ feelings of personal and team pride,^3^^,^^7^^,^^32^ plus development of athlete identity^3^^,^^31^^,^^33^^,^^40^ (Table 2, Table 3). The most commonly-reported benefits by BCS from qualitative and mixed-methods studies were increased social support (e.g., peer and family support; n = 12) and posttraumatic growth (e.g., regaining control over their bodies and lives, cultivating new possibilities and purposes, increased personal/emotional strength, increased confidence in navigating future challenges, being able to move on and accept cancer diagnosis, appreciation of life; n = 11) (Table 4, Table 5).

However, the benefits of dragon boating on psychosocial outcomes were less consistently demonstrated in quantitative studies/mixed-methods study. Among the 7 quantitative/mixed-methods studies measuring psychosocial outcomes, mixed findings were apparent in the benefits of quality of life (4 studies showing significant improvements^24^^,^^26^^,^^29^^,^^43^ and 2 did not^23^^,^^25^) and body image (1 study showing significant improvements^44^ and 1 did not^23^). However, one quasi-experimental study in Italy^44^ found that BCS in the dragon boating group reported significantly fewer traumatic stress symptoms than the control group (engaging in alternative physical activities). Overall, the impacts on psychosocial well-being were more likely to be reported in qualitative studies (Table 3, Table 4).

Among the 3 studies (all qualitative) reporting impacts on health behaviors, BCS perceived a need to eat well and stay fit for racing^32^ and reported an increased awareness in healthier lifestyle (e.g., diet, exercise, stress management).^33^^,^^43^ No quantitative studies evaluated BCS’ changes in health behaviors (Table 4).

### 3.6 Research question 2: Potential facilitators and barriers related to dragon boating participation

**Fig. 4:**
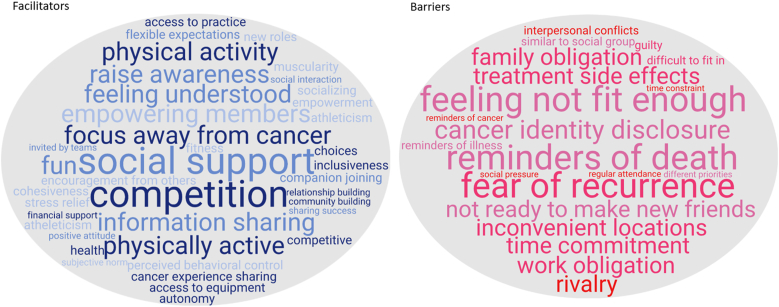
Word clouds illustrating the common facilitators and barriers of participating in dragon boating exercise programs *Note*: Word sized according to the proportion of responses apparent in the themes.

Ten studies (7 qualitative and 3 quantitative) investigated the facilitators and barriers related to BCS’ participation in dragon boating exercise (Table 6). The common facilitators and barriers were also visually presented by the word clouds in Fig. 4. Common facilitators included social support/social connection, focus away from cancer, information sharing, being physically active and competitive; common barriers included not feeling fit enough to participate, concerns about cancer identity disclosure, time commitment, and location constraints.

**Table 6:** Facilitators and barriers related to breast cancer survivors’ participation in dragon boating exercise (n = 10).

	First author, year (region)	Design	Sample characteristics and dragon boating experience	Facilitators and barriers of participation
1	McDonough, 2019 (USA)	Qualitative	17 BCS: Attended at least one practice of a newly-formed dragon boating team	Facilitators: - Seeing benefits of gaining social support, health, having fun - Seeing dragon boating as an accessible activity to be active and competitive - Opportunity to focus away from breast cancer - Joining the team with someone they knew - Encouragement from friends, family members, health professionals - Opportunity to raise awareness about breast cancer/community building Barriers: - Concerns about physical capabilities hindering participation - Feeling accountable to teammates (regular attendance needed) - Inconvenient locations and time constraints - Feeing guilty taking up a space for others needing more support - Bringing up personal fears of recurrence - Concerns about difficulties to integrate into a group - Work and family obligations - Not wanting to be publicly identified as breast cancer survivors - Not interested in rivalry/competitiveness of dragon boating
2	Weisenbach, 2014 (USA)	Qualitative	15 BCS having decided to join a dragon boating team	Facilitators: - Opportunity for fun, excitement, physical activity, and social interactions - Flexible expectations about attendance - Uplifting and empowering attitude of dragon boat team members - Personal invitations from team members making survivors feel wanted/included Barriers: - Concerns about side-effects of treatments making paddling difficult - High time demands and distance to practice - Not wanting to be too visible to the community - Discomfort with initiating new social connections - Similarity to a support group (sick of talking about breast cancer) - Feeling social pressure when receiving invitations to join
3	Unruh, 2004 (Canada)	Qualitative	3 BCS: Members of a dragon boat team Involved with dragon boat racing within 6 months to 3 years following diagnosis	Facilitators: - Being with other women sharing cancer experiences - Enthusiasm and positive energy of other women - Competition as the motivation to change and experience pride - Implicit support without explanation - Opportunity to increase public awareness and perceptions of breast cancer Barriers: - Competition discouraging women who did perceive themselves as athletic - Competition causing divisions within the team - Recurrence or death of team members triggering fears
4	Sabiston, 2007 (Canada)	Qualitative	20 BCS: Members of dragon boat teams At least their second year of participating in dragon boat program	Facilitators: - Opportunity to cope with stress - Opportunity to receive and provide social support Barriers: - Constant reminders about cancer - Needing to deal with others' illness and death - Being too visible to the public about survivor identity
5	McDonough, 2008 (USA)	Qualitative	14 BCS: Members of dragon boating program First year of participation Interviewed two times	Facilitators: - Comfortable contexts feeling at ease with their bodies (all shapes and sizes in the same boat) - Implicit understanding of breast cancer experience - Opportunity to share first-hand information about breast cancer experience - Providing a context where breast cancer was not the focus Barriers: - Reminders about being a cancer survivor - Reminders of the possibility of recurrence and death - Not wanting to establish social connections with teammates due to survivor identity
6	McDonough, 2011 (USA)	Qualitative	17 BCS: Members of a dragon boat team Attended at least one practice Interviewed four times	Facilitators: - Feeling empowerment and encouragement from the teammates - Implicit understanding of breast cancer experience - Opportunity to share first-hand information about breast cancer experience|- Motivated to pursue fitness, athleticism, competition Barriers: - Interpersonal conflicts with teammates - Differences in priorities about performance/race - Reminders of the possibility of recurrence and death - Work and family obligations - Perceived lack of fitness and sport competence - Lack of time - Concerns about side-effects of treatments
7	Hadd, 2010 (Canada)	Quantitative	470 BCS: on average 3.06 years in dragon boating Participating in an international dragon boating competition	Facilitators: - Participating in dragon boating/physical activity being viewed as positive - Forming relationship with other survivors being viewed as positive - Feeling of increased muscularity being viewed as positive
8	Culos-Reed, 2005 (Canada)	Quantitative	109 BCS from six breast cancer dragon boat teams Participating a competitive season of dragon boating	Facilitators: - Perceived behavioral control was concurrently associated with intention to participate in training (Time 1 & Time 2) - Attitude towards dragon boat training at the early season (Time 1) was prospectively associated with intention to participate in training 12 weeks later (Time 2)
9	Courneya, 2001 (Canada)	Quantitative	24 BCS: Members of a dragon boating racing team Attending a twice weekly, 12-week training program	Facilitators: - Subjective norm was associated with higher intention to attend the program - Intention was associated with higher program attendance
10	Fong, 2021 (Canada)	Qualitative findings (from a mixed-methods study)	9 BCS (in a sample of 11 current/former coaches from 7 dragon boat teams): Active paddlers on the teams	Strategies to facilitate quality participation: - Autonomy to make choices about practices and voice opinions - Opportunity to be social as a team inside and outside of sport-related activity - Personalized training programs and friendly competition - Encouraging survivors to participate in other physical activities - Sharing athletes' success through social media - Encouraging survivors to see themselves as athletes - Facilitating travel, access to practice/regattas, access to equipment - Offering opportunities to offset athletes' costs (e.g., fundraising) - Encouraging athletes to take on new roles - Cohesive group environment

### 3.7 Facilitators

It is common for BCS to see dragon boating as opportunities for social connection and support network.^32^^,^^45^^,^^46^ They appreciated the opportunities to connect with peers who shared similar cancer experiences, and to provide support (sometimes implicit) and information mutually. The group environment, often characterized by enthusiasm and empathy, helped BCS feel included and uplifted.^47^^,^^48^ On the other hand, BCS in some studies believed that dragon boating allowed them to have fun/excitement, to stay active, and to focus less on breast cancer.^49^, ^50^, ^51^ The opportunity for competition was also regarded as the motivation to change and experience pride (Table 6).^32^

Theory-based psychosocial variables also contributed as facilitators. Among the two studies applying the Theory of Planned Behavior, Courneya and colleagues^49^ found that subjective norms significantly contributed to BCS’ intention to engage in dragon boating training, whereas intention was the primary factor of exercise adherence. Conversely, Culos-Reed et al.^50^ found that perceived behavioral control and attitude towards dragon boating, respectively, were associated with current and future intentions to practice. In line with those findings, it is also noteworthy that encouragement from family, friends, health professionals, and even invitations from current team members helped reduce hesitations in joining (Table 6).^45^^,^^47^

### 3.8 Barriers

However, BCS also experienced barriers for their participation in dragon boating. First, personal concerns over physical limitations reduced their commitment to dragon boating exercise (e.g., treatment side-effects, perceived lack of fitness).^45^^,^^47^^,^^48^ Second, conflict within teams, differences in commitment levels, and discomfort in forming new connections were identified as challenges.^47^^,^^48^ Third, logistical constraints(e.g., high time commitment, inconvenient locations) and challenges in other life domains(e.g., work/family obligations, financial concerns) also contributed to lower adherence to dragon boating exercise (Table 6).^45^^,^^51^

Some BCS found the competitive aspect of dragon boating off-putting, especially among those uninterested in rivalry or those believing that it undermined group cohesion.^32^ The ambivalence over survivor identity visibility was also apparent. While some BCS felt empowered by raising awareness and forming community, others were reluctant to disclose their cancer survivors identity publicly or to constantly be reminded of their cancer experiences.^31^^,^^33^ Besides, dragon boating might be viewed as a reminder of death/cancer, particularly when members in the team experienced recurrence or even passed away^32^^,^^48^ (Table 6).

Furthermore, one qualitative study^51^ interviewed 11 current/former leaders from seven dragon boat teams in Canada (including 9 female BCS) to provide insights in the potential strategies for addressing barriers related to dragon boating for BCS. It underscored the importance of autonomy, allowing paddlers to make decisions about their practices and team dynamics. Moreover, providing individualized training programs, encouraging BCS to participate in other physical activities, organizing friendly competition could also allow BCs to see themselves as athletes. These strategies could help BCS feel empowered, motivated, and respected within the team structure. Other supportive structures and logistics (e.g., facilitating access to regattas and equipment, offsetting costs through fundraising, using social media to share team success, social gatherings outside the boat) were also important strategies to reduce BCS’ barriers to participate in dragon boating exercise (Table 6).

## 4 Discussion

Extending from existing reviews,^8^^,^^9^ this was the first review synthesizing the available evidence on *both* the impacts of dragon boating exercise on BCS’ multiple domains of well-being and the factors associated with participation in dragon boating exercise.

### 4.1 Impacts on well-being

Among the 27 studies addressing RQ1, 24 studies reported at least one benefit on well-being. Such benefits are apparent across different types of studies (including RCTs, quasi-experimental, single-trials, and observational studies). Specifically, our review indicated that a high proportion of studies demonstrated benefits of dragon boating exercise in at least one indicator for physical functioning (15 out of 17 studies, e.g., pain, fatigue, dyspnea, insomnia, shoulder functioning, cardiorespiratory function) among BCS, with improved (upper/lower) limb strength as more robust benefits observed. Improvements in biochemical/physiological indicators (4 out of 6 studies) were also apparent in the reviewed studies, although the significantly improved indicators differed across the studies. However, we should be cautious when interpreting the results given the variations in measurement scales, study design, sample size involved (some even having sample sizes ≤50 participants), and the comparison groups might contribute to the statistical significance of the study findings.

It is noteworthy that studies consistently found that engaging in dragon boating did not increase the risk of lymphedema. It was consistent with a systematic review of 6 RCTs examining the impacts of resistance exercises on increasing muscular strength without increasing lymphedema risks.^52^ These findings suggested that dragon boating could be a safe alternative for physical activity for BCS. It challenged clinical guidelines that women with axillary dissection were to refrain from engaging in vigorous, repetitive, or excessive upper body exercise.^28^

Most of the evidence on the impacts on psychosocial well-being was from qualitative studies, with BCS members of dragon boat teams being interviewed. BCS paddlers were metaphorically and literally all “in the same boat”.^43^ The team-based nature of dragon boating provides a supportive and inclusive environment which facilitates information sharing, building networks with fellow survivors, and developing self-confidence. That is why those qualitative studies consistently found that dragon boating is beneficial to BCS in enhancing their stress and coping skills,^30^, ^31^, ^32^^,^^40^ post-traumatic growth,^31^^,^^34^^,^^48^ appreciation of life,^34^^,^^41^ perceived strength and fitness,^33^ plus awareness in healthier lifestyle (e.g., diet, exercise, stress management).^33^^,^^43^

There were fewer intervention or quantitative observational studies evaluating the impacts of dragon boating on psychosocial well-being and changes in health behaviors among BCS; the findings were less conclusive. For example, BCS dragon boat intervention group reported more improvement in body image in one study (compared against home-based exercise group),^23^ but not the other (compared against other exercises of personal preference).^44^ Inconsistent findings were also found in the impacts on overall quality of life.^23^^,^^29^

Based on the current state of evidence, most studies have demonstrated that dragon boating is generally beneficial to BCS’ multiple domains of well-being(i.e, physical functioning, biochemical indicators, psychosocial well-being, and health behavior change). Stakeholders of intervention programs (including researchers, healthcare professionals, BCS) should be aware of the benefits of dragon boating as an alternative rehabilitation tool for BCS. However, the strength of evidence did not sufficiently support that dragon boating is a better option than other physical activities. A prior review suggested that physical activity interventions involving moderate-to-vigorous exercise with 150 min for 3 times per week and in any modality might provide better health outcomes among BCS during adjuvant therapy.^4^ Given that the frequency, duration, intensity, format of dragon boat programs, and the health outcomes measured varied across studies, the heterogeneity of current evidence makes it difficult to estimate what dosage of intervention in dragon boating programs would be sufficient for BCS to reap the benefits in different domains of well-being. Another important observation was that many of the included studies did not clearly state the details about the dragon boating exercise programs in terms of their duration, frequency per week, and time spent per session. Future studies should better document the details of the interventions and examine the optimal dosage of dragon boat exercise for BCS’ well-being through more robust intervention designs(e.g., RCTs).

### 4.2 Facilitators and barriers associated with BCS’ participation in dragon boating

Another important and unique contribution of this review was the exploration of the facilitators and barriers of BCS’ participation in dragon boating. The major facilitators for BCS to participate in dragon boating included: 1)social support/social connection, 2) seeing dragon boating as opportunities to focus away from cancer, 3)information sharing, and 4)being physically active and competitive.

Those facilitators are similar to those identified to increase engagement of physical activity among BCS (e.g., expected health benefits, supportive environment, feeling of empowerment) from a review of 13 studies.^11^

From quantitative studies, we also found that variables from the Theory of Planned Behavior(e.g., attitudes, subjective norms, perceived behavioral control, behavioral intention) were useful in explaining BCS’ intention to participate in training and actual adherence to training. Highlighting those factors when introducing the sport to BCS might increase their BCS’ willingness to start and maintain training.

Several barriers were prevalent among the BCS. Given that dragon boating involves paddling in a large and heavy boat, it could be challenging and potentially dangerous for individuals who are not familiar with the equipment or techniques. Some BCS may believe that physical limitations or side-effects from cancer treatment make them inappropriate for dragon boating. Similar concerns have also been apparent among BCS participating in other physical activities.^11^ Tailor-making training programs might be needed at the initial stage of training could be helpful.

Second, from an implementation perspective, factors related to dragon boating-specific logistics should also be considered. For example, the availability of suitable water bodies may be limited in some areas/regions, which may make it difficult to establish dragon boating programs. In addition, transportation to and from the water body and the equipment can be a challenge, especially for those who live far away or have limited access to transportation.^51^ Those challenges have also been discussed in a review on nature-based watersport interventions (e.g., kayaking, surfing, etc.) for individual dealing with a range of health issues.^53^

Besides, dragon boating programs typically require regular weekly practices, which can be difficult to fit into busy schedules.^45^^,^^54^ Thus, it might be crucial to address the barrier regarding time commitment required for dragon boating, particularly those BCS who are still working or have caregiving responsibilities.^55^ Dragon boats are specialized equipment that may require significant financial investment (for storage and maintenance), which may be a challenge for some organizations or individuals.^55^^,^^56^ Therefore, support in structures and logistics(e.g., facilitating access to regattas and equipment, reducing financial burden through fundraising, using social media to share team success, social gatherings outside the boat) could be helpful strategies to reduce BCS’ barriers to participate in dragon boating exercise.^51^

A few factors function as double-edged swords in facilitating/hindering BCS’ participation in dragon boating. First, even though BCS could enjoy the benefits of gaining social support and engaging in social interactions with other survivors, some might experience interpersonal conflicts with teammates due to differences in priorities. Such findings were also aligned with prior reviews showing that group-based exercise interventions did not always provide additional benefits over individual-based exercise interventions for BCS.^57^^,^^58^ Second, BCS appreciated that dragon boating allowed them to focus less on breast cancer,^33^^,^^45^ but the cancer recurrence or death of team members could trigger fear and grief responses.^31^, ^32^, ^33^^,^^48^ Dragon boat team managers and coaches might need to provide additional interventions to support team members’ emotional needs and help them go through the grief process.^32^ Third, the ambivalence over visibility of survivor identity was common among BCS. Some BCS felt empowered by the mission to raise public awareness about breast cancer,^32^^,^^45^ but others were not willing to be too visible to the public about their survivor identity.^31^^,^^47^ Given that self-stigma is still prevalent among BCS,^59^ their levels of readiness to be visible in public could differ. Such issues should also be properly addressed when designing dragon boat programs for BCS. It will also be important for future studies to examine if dragon boating exercise programs addressing those facilitators and barriers will be more accepted by BCS and more effective in improving their different domains of well-being.

## 5 Limitations

This review was subject to several limitations. First, there were potential publication biases. Scoping reviews could be susceptible to publication bias, as they typically rely on published literature rather than unpublished studies or gray literature. Findings of the review may be influenced by the availability and quality of published studies on the topic. Second, given that the dosage, intensity, and research design of the dragon boat exercises program differ across studies (Table 1), making a systematic comparison among those studies not possible. Therefore, the analysis of the current evidence was limited, which might not be able to examine the relationships between different studies or the strength of the evidence comprehensively. Third, even dragon boating was originated in China, using it as a tool for rehabilitation among postoperative BCS started from Western countries (e.g., Canada, the United States, Italy) (Fig. 2). That might be the reason why we could only identify a few studies conducted in Asian regions addressing this topic in health settings. The applicability of findings to non-Western cultural contexts has yet to be further examined.

## 6 Conclusion

Based on our review on current findings, the evidence on the physical and psychosocial benefits of dragon boating for breast cancer survivors seems convincing. Dragon boating could be a favorable option of physical activity for BCS. For future directions, researchers should examine the optimal dosage of dragon boat exercise for BCS’ well-being through more robust intervention designs(e.g., RCTs) and how the benefits from dragon boating exercise are comparable to/unique from other physical activity programs (e.g., rowing, yoga, strength training, walking, etc.). To translate such findings into actual benefits among BCS, it is equally important to consider these potential facilitators/barriers and develop strategies to address them when planning, promoting, and implementing dragon boating programs in the community settings.

## Prior presentation

Part of the findings were presented at the 1st Chinese Physical Literacy Association International Conference, Hong Kong, China.

## Author contributions

N.Y. conceptualized the study, conducted literature reviews, overseeing the searches and tabulation of data, conducted data analysis, wrote the first draft of the manuscript, and finalized the manuscript. V.T. and S. L. conducted literature reviews, tabulated the data, conducted data analysis, and supported the drafting and finalization of the manuscript. L.*P. and* S.N.N supported the processes of searches and managements of selected articles. D.Y.S. and R.S. contributed to the discussion section, reviewed, and edited the manuscript. All authors approved the final version of the manuscript.

## Data and resource availability

The datasets generated during and/or analyzed in the current study are available from the corresponding author upon reasonable request.

## Funding sources statement

The initiation of the project was supported by The Taiwan Collaboration Fund (2019–2020, PI: Nelson Yeung), The 10.13039/501100004853Chinese University of Hong Kong.

## Conflict of interest statement

Author Raymond Kim Wai Sum is a member of the Editoral Board of Journal of Exercise Science and Fitness. Raymond Sum was not involved in the journal's peer review process of, or decision related to, this manuscript. The other authors have no conflicts of interest relevant to this article.
